# Nanoplasma Formation by High Intensity Hard X-rays

**DOI:** 10.1038/srep10977

**Published:** 2015-06-16

**Authors:** T. Tachibana, Z. Jurek, H. Fukuzawa, K. Motomura, K. Nagaya, S. Wada, P. Johnsson, M. Siano, S. Mondal, Y. Ito, M. Kimura, T. Sakai, K. Matsunami, H. Hayashita, J. Kajikawa, X.-J. Liu, E. Robert, C. Miron, R. Feifel, J. P. Marangos, K. Tono, Y. Inubushi, M. Yabashi, S.-K. Son, B. Ziaja, M. Yao, R. Santra, K. Ueda

**Affiliations:** 1 Institute of Multidisciplinary Research for Advanced Materials, Tohoku University, Sendai 980-8577, Japan; 2 Center for Free-Electron Laser Science (CFEL), DESY, 22607 Hamburg, Germany; 3 The Hamburg Centre for Ultrafast Imaging, 22761 Hamburg, Germany; 4 RIKEN SPring-8 Center, Sayo, Hyogo 679-5148, Japan; 5 Department of Physics, Kyoto University, Kyoto 606-8502, Japan; 6 Department of Physical Science, Hiroshima University, Higashi-Hiroshima 739-8526, Japan; 7 Department of Physics, Lund University, P.O. Box 118, 22100 Lund, Sweden; 8 Blackett Laboratory, Imperial College London, London SW7 2AZ, United Kingdom; 9 Synchrotron SOLEIL, L’Orme des Merisiers, Saint-Aubin, BP 48, FR-91192 Gif-sur-Yvette Cedex, France; 10 Extreme Light Infrastructure - Nuclear Physics (ELI-NP), “Horia Hulubei” National Institute for Physics and Nuclear Engineering, 30 Reactorului Street, RO-077125 Măgurele, Jud. Ilfov, Romania; 11 Department of Physics and Astronomy, Uppsala University, P.O. Box 516, SE-751 20 Uppsala, Sweden; 12 Department of Physics, University of Gothenburg, SE-412 96 Gothenburg, Sweden; 13 Japan Synchrotron Radiation Research Institute (JASRI), Sayo, Hyogo 679-5198, Japan; 14 Institute of Nuclear Physics, PAS, Radzikowskiego 152, 31-342, Krakow, Poland; 15 Department of Physics, University of Hamburg, 20355 Hamburg, Germany

## Abstract

Using electron spectroscopy, we have investigated nanoplasma formation from noble gas clusters exposed to high-intensity hard-x-ray pulses at ~5 keV. Our experiment was carried out at the SPring-8 Angstrom Compact free electron LAser (SACLA) facility in Japan. Dedicated theoretical simulations were performed with the molecular dynamics tool XMDYN. We found that in this unprecedented wavelength regime nanoplasma formation is a highly indirect process. In the argon clusters investigated, nanoplasma is mainly formed through secondary electron cascading initiated by slow Auger electrons. Energy is distributed within the sample entirely through Auger processes and secondary electron cascading following photoabsorption, as in the hard x-ray regime there is no direct energy transfer from the field to the plasma. This plasma formation mechanism is specific to the hard-x-ray regime and may, thus, also be important for XFEL-based molecular imaging studies. In xenon clusters, photo- and Auger electrons contribute more significantly to the nanoplasma formation. Good agreement between experiment and simulations validates our modelling approach. This has wide-ranging implications for our ability to quantitatively predict the behavior of complex molecular systems irradiated by high-intensity hard x-rays.

The interaction between matter and intense laser fields is of fundamental interest to physics. This interest is also strongly driven by more applied fields, such as x-ray imaging of biological samples^1^. During imaging the dynamics within an x-ray irradiated sample is strongly affected by photoinduced ionization^2^. Atomic clusters in vacuum are ideal study objects for investigating this in detail, not only because their size can be varied from a single atom to mesoscopic scales but additionally there is no energy dissipation from clusters into a thermodynamic bath^3^,^4^.

So far, the interaction of rare-gas clusters with strong near infrared (NIR) laser pulses has been studied in most detail^5^,^6^. In strong NIR laser fields, the electromagnetic field ionizes the sample via field ionization. A nanoplasma is formed when the electrons ejected from atoms in an ionization process remain trapped by the Coulomb potential of the multiply charged cluster ion (‘inner ionization’). The trapped electrons further acquire energy from the laser field via inverse bremsstrahlung (IBS) and then cause secondary ionizations^7^. When the electrons gain sufficient energy, they escape from the cluster (‘outer ionization’). When the laser field is turned off and the nanoplasma expands, electrons trapped in the nanoplasma recombine with individual atomic ions^8^,^9^,^10^,^11^,^12^.

Rare-gas clusters exposed to strong extreme ultraviolet (EUV) laser pulses have also been studied extensively^3^,^4^,^13^,^14^,^15^,^16^ with the advent of EUV free-electron lasers (FELs)^17^,^18^. In the EUV regime, the main mechanism of cluster ionization and heating is single-photon absorption of the individual atoms in the cluster; IBS heating is negligible. With increasing charge of the cluster, photoelectrons are decelerated and eventually get trapped; inner ionization still proceeds. As the number of trapped electrons increases, they are thermalized through collisions and a nanoplasma is formed. Efficient nanoplasma formation and thermal emission from nanoplasma is observed when the photon energy is set below the ionization threshold of the individual atom, or through multiple excitation to the conduction band (equivalent to inner ionization)^19^ (see Ref. [20] for the related phenomenon of thermionic emission).

Recently, the experimental observations of rare-gas clusters have been extended to soft x-ray photon energies of ~500 eV (argon^21^, size up to ~50 Å) and ~800 eV (xenon^22^,^23^, size >100 Å) at the Linac Coherent Light Source, an x-ray FEL (XFEL) facility in the U.S.^24^. Under those conditions L-shell electrons of argon atoms could be directly photoionized, ejecting photoelectrons with a kinetic energy ~230 eV, whereas xenon could be photoionized in the M shell, ejecting electrons with an energy ~130 eV. In both cases the inelastic mean free path of the photoelectrons in the sample (~15 Å) is smaller than the size of the clusters. Therefore those photoelectrons predominantly contribute to the nanoplasma formation by generating low energy electrons during collisional ionization.

In the present work, we have investigated experimentally argon and xenon clusters with average size of up to 1000 atoms irradiated by focused XFEL pulses at ~5 keV. The experimental results are compared quantitatively to and interpreted through our detailed simulations. In this high photon energy regime new photoionization channels and, therefore, new intra-atomic decay channels are open.

In the argon case even K-shell photoionization can occur and the K hole is filled typically through Auger decay. The ejected photo- and KLL/KLM/KMM Auger electrons are energetic (>1.5 keV) and likely to escape from the cluster without generating other free electrons via inelastic collisions with the ions (inelastic mean free path 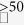 Å, cluster radius 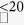 Å). Therefore these primary electrons can be expected not to contribute to the nanoplasma formation, in contrast to ‘photoionization heating’ using soft x–rays^22^,^25^. The energy of an absorbed photon is mainly transferred to the photoelectron and the high energy Auger electron and only a small part of the energy, ‘stored’ in the excited ion, is transferred to the plasma via LLM and LMM Auger events. These decays release slow electrons that can be trapped within the cluster and cause secondary ionizations. The ionization cascades distribute the energy of the Auger electron and finally lead to the nanoplasma formation. In this way a highly indirect plasma formation occurs: neither the electrons are originating nor the energy of the plasma is transferred in a direct way from the photon absorbed, but via secondary processes following the photoabsorption process. This scenario may be expected to be typical for biological systems of medium size, irradiated under imaging conditions, i.e., when the photon energy is much higher than all atomic binding energies. Nanoplasma electrons then do not only modify the dynamics of the ions but the scattering signal from nanoplasma electrons also contributes significantly to the high resolution diffraction patterns^26^,^27^,^28^,^29^.

In the Xe clusters, at the photon energy used, the photoelectrons are ejected primarily from the L shell (rather than from the M shell^22^,^23^). This has the following consequences. First, the photoelectrons are relatively slow (<700 eV). Second, the number of possible electronic configurations is extremely high, thus making theoretical modelling much more complex and challenging.

Our experimental results obtained at SPring-8 Angstrom Compact free electron LAser (SACLA)^30^ in Japan provide evidence for nanoplasma formation by hard x-ray radiation. Moreover, our theoretical modelling accurately reproduces the significant trends in the experimental observations and reveals further details of the dynamics within the irradiated clusters. The current study also serves as a validation of our modelling approach, based on electron spectroscopy data, complementing an earlier work^31^ based on ion data. Our approach does not involve any parameter fitting for describing the ionization and real space dynamics of the system.

Figure 1 depicts experimental and theoretical electron spectra from Ar clusters irradiated by the focused XFEL pulses. The average sizes of the clusters (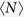) were estimated to be ~100, ~300, and ~1000, using the scaling law^32^,^33^. All the spectra show two main characteristic features. The broad peak at ~200 eV observed for relatively small cluster size (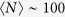) corresponds to the L_2,3_MM Auger emission from the (charged) clusters (atomic lines are between 100–300 eV, Fig. 1 (d)). With increasing the cluster size, a stronger positive electrostatic potential builds up. The deceleration of the electrons ejected later in the pulse is then larger, producing a stronger signal towards lower energies in the spectra. This finally turns the valley near 50–100 eV into a plateau feature^8^,^14^ for large clusters (Ar_300_, Ar_1000_ in Fig. 1). The decelerated continuous L_2,3_MM Auger electron emission is analogous to the decelerated continuous photoelectron emission from clusters irradiated by EUV-FEL pulses^14^,^16^. The other characteristic feature – the strong peak at zero kinetic energy – can be interpreted as the thermal electron emission from the nanoplasma, based on the earlier EUV-FEL studies^14^,^19^,^25^,^34^.

The calculated spectra capture the significant trends in the experimental result and agree well for large (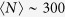, 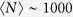) clusters confirming the validity of our theoretical model. For small clusters (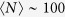) we found that we could obtain a better agreement with experiment by increasing the relative weight of lower fluence regions in the volume integrated spectra (not shown). That could have been caused in the experiment by, e.g., a halo which may exist around the focused beam (see Supplementary Information). Another uncertainty arises (especially for small clusters) from the uncertainties of the cluster size: the one based on the scaling law^32^,^33^ of the cluster size distribution and the one due to its possible spatial dependence, which would affect volume integrated signals.

In the following paragraphs we give more insight into the evolution of the clusters based on Ar_1000_ systems. In Fig. 2 the dynamics of Ar_1000_ in the focus of the x-ray beam is illustrated by the trapping potential of the cluster at different times. The positive electrostatic potential of the cluster is responsible for the nanoplasma formation. The inset of Fig. 2 shows the corresponding electron spectra. During the pulse, only the plateau is being formed, which is mostly due to the slowed down LMM Auger electrons. The main peak at 0 eV develops during several hundreds of femtoseconds after the pulse. The ionic system then starts to expand, the cluster potential becomes shallower and lets some of the trapped electrons escape. The time separation of the peak formation from the x-ray pulse gives a clear evidence that this peak originates from the thermal emission from the nanoplasma and is not due to electrons leaving the cluster directly after the photoionization events. Although the plasma peak is a feature of time and volume integrated experimental data, we were still able to fit it with an exponential to extract a characteristic temperature value. Such a fit yields an electron temperature of ~5 eV. Theoretical modelling gives average kinetic energies of ~3–8 eV in the period between ~0.5–1.5 ps after the pulse (when plasma emission in Ar_1000_ in the focus occurs in the simulations), i.e., in good agreement with the experiment.

Further details of the electron emission are revealed from the theoretical studies (Fig. 3). The contribution of high energy electrons (>2 keV; outside the detection range of our experiment) to nanoplasma formation via secondary ionizations is small due to the small impact ionization cross sections. This negligible contribution is reflected by the shape of the high energy photoelectron and Auger peaks in the volume integrated spectrum calculated theoretically for the entire available energy range, 0–5 keV. The peaks broaden only due to the growing trapping potential of the cluster^35^.

Modelling also enables us to extract detailed quantitative information on the relative contribution of each ionization process. When a 1000-atom cluster experiences 50 *μ*J/*μ*m^2^ fluence, which corresponds to that in the beam focus, approximately 16.5% of the atoms absorb a photon, in ~90% of these events by core electron ejection. The core hole is quickly filled up due to KLL, KLM or KMM Auger decays or fluorescent relaxation with branching ratios of 75.5%, 13.5%, 0.2% and 10.8%, respectively. Inner shell cascading continues further within these atoms by LLM (~0.25 events per atom) and LMM (~1.75 events per atom) Auger decays. While inner shell relaxation happens to photoionized atoms only, impact ionizations occur in neutral atoms as well. The average number of impact ionizations is ~2 events per Ar atom. Only approximately 1% of impact ionizations are caused by high energy photoelectrons and KLL, KLM Auger electrons. The majority (~90%) of trapped electrons are created by impact ionizations caused by low-energy Auger and secondary electrons. The total ionization degree of ~2.6 per atom is reached during and shortly after the irradiation. However, within 1 ps the average charge decreases already by ~40% due to recombination.

The Xe clusters were irradiated under similar conditions: the x-ray parameters were the same as for the Ar case except the photon energy was slightly larger (5.5 keV). The basic features of the spectra (plateau and plasma emission peak, Fig. 4) are similar to the Ar case. There is a good agreement between experiment and modelling. However, at low energies a more significant discrepancy can be seen. Beside the pulse intensity profile and cluster size uncertainties, another possible source of this deviation is that photo- and Auger electron energies for heavy elements by XATOM are less accurate. Currently, a non relativistic theory is used in the code and the omission of spin-orbit coupling introduces binding energy shifts that may be reflected in the calculated electron spectra. Better agreement may then be achieved once a relativistic version of XATOM becomes available.

In deep-shell ionized xenon, intra-atomic Auger cascades are built up from many sequential Auger emission steps. In a 300-atom system experiencing the maximum fluence in the beam, 1 photoionization, 4 Auger decay and 2.75 secondary ionization events per atom occur on average. Therefore the ionization degree is much larger than in Ar. Among the most probable processes only LMM Auger decay leads to electrons energetic enough (>3 keV) to escape without relevant participation in plasma formation, representing a small fraction (~7%) of all ejected electrons. Other statistically relevant Auger and photoionization processes generate electrons typically around 700 eV, 400 eV and 30 eV, which contribute to the plasma formation. Our study shows that the relative contribution of secondary ionization to plasma formation is smaller in systems composed of heavy elements irradiated by hard x–rays due to the multi-step Auger cascades.

In summary, we have investigated nanoplasma formation from argon and xenon clusters irradiated by intense x-ray pulses at ~5 keV, using the new XFEL facility SACLA as well as our newly developed modelling tool, XMDYN. The simulation of xenon clusters is the first demonstration of a novel methodological development connecting the codes XMDYN and XATOM on-the-fly. This enables high-accuracy modelling of systems containing strongly (deep-shell) ionized heavy atoms. The good agreement between experiment and theory serves as an important validation step of the modelling approach. We identified that a nanoplasma is formed efficiently within the clusters by trapping low-energy electrons. It is formed already during the x-ray pulse of 10 fs duration but the following thermal electron emission lasts long after the pulse is finished. Nanoplasma formation by trapping of low-energy secondary electrons is of universal occurrence. Thus, understanding this mechanism in detail is also crucial for other research areas with XFELs, such as femtosecond x-ray imaging^1^,^36^,^37^,^38^, where the electronic radiation damage, especially to heavy atoms, is of high importance.

## Methods

The experiment has been carried out at the experimental hutch 3 (EH3) of beamline 3 (BL3)^39^ at SACLA in Japan. The experimental configuration is described in the Supplementary Information. In brief, the x-ray source produced pulses of 5 keV (in case of Xe 5.5 keV) photons with 10 fs estimated duration. The rare-gas clusters were prepared by adiabatic expansion of the rare gas (Ar or Xe) through a 250 *μ*m nozzle at room temperature. The stagnation pressures were 0.43, 0.67 and 1.12 MPa for Ar and 0.14 and 0.21 MPa for Xe, and the averaged cluster size 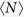 were estimated to be ~100, ~300 and ~1000 for Ar and ~100 and ~300 for Xe, respectively, according to the well-known scaling low^32^,^33^. If we use more recent work^40^ for size estimation, the estimated cluster size becomes ~20% smaller for the cluster size of ~100, but does not change for the larger sizes. The interaction volume, where the XFEL pulse interacts with the cluster beam, had roughly a cylindrical shape of ~1 *μ*m in diameter (i.e., XFEL focus size) and ~2 mm (i.e., the cluster beam diameter) along the XFEL beam. A maximum of ~50 *μ*J/*μ*m^2^ fluence (~5 × 10^17^ W/cm^2^ peak intensity) was reached in the focus. Electrons produced in this interaction volume were extracted towards a velocity map imaging (VMI) spectrometer^41^,^42^,^43^. The spectrometer employed in the present experiment was specifically designed for use at the XFEL facility so that high energy electrons up to 1 keV could be detected. Brief descriptions of the spectrometer and the analysis procedure specific to this novel high-energy VMI are also given in the Supplementary Information. With this spectrometer, we have recorded the electron spectra in the energy range from zero to 320 eV for Ar and to 960 eV for Xe.

For the theoretical modelling, we employed XMDYN^44^, our tool developed for modelling finite systems such as clusters irradiated by XFEL. XMDYN has been successfully applied for modelling small molecules (C_60_) irradiated by high intensity XFEL^31^ and low–intensity synchrotron^45^ pulses. It combines a Monte Carlo description of ionizations with the efficient classical molecular dynamics treatment of particle dynamics (atoms, atomic ions and free electrons). Photoionization, inner shell relaxations (corresponding cross sections and rates are calculated with the XATOM code^46^) and electron impact ionizations (via the atomic binary-encounter-Bethe (BEB) cross sections^47^) are included. XATOM^46^ has been already successfully applied for theoretical modelling of rare gas atoms irradiated by SACLA^48^,^49^.

The theoretical description of strongly ionized heavy elements is very challenging due to the very large number of available electronic configurations and decay channels (>2 × 10^7^ and 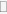
10^8^, respectively for Xe with L-shell dynamics included)^49^. While one still can pre-calculate and tabulate cross sections and rates for all electronic states of Ar, this is computationally not feasible for Xe. Instead, we made an on-the-fly link between the XATOM and XMDYN codes so that the relevant parameters are calculated for electronic states that appear during one Monte Carlo realization. To our knowledge this is the first time that such accurate, configuration-sensitive calculations were carried out for clusters consisting of strongly ionized heavy elements. This methodological development is of high importance as it constitutes a high-accuracy scheme for modelling irradiation of samples containing heavy elements.

The XFEL pulse intensity distribution is spatially non-uniform within the XFEL beam and within the interaction volume defined by the intersection of the beam and the jet. Therefore the measured signal contains contributions from a wide range of fluences. They were included in the calculations (see Supplementary Information). Further, we took into account the experimental log-normal distribution of the cluster size. However, size averaging does not introduce significant changes in the spectra. The trajectories were propagated up to 1.5 ps after the pulse.

## Additional Information

**How to cite this article**: Tachibana, T. *et al.* Nanoplasma Formation by High Intensity Hard X-rays. *Sci. Rep.* **5**, 10977; doi: 10.1038/srep10977 (2015).

## Supplementary Material

Supplementary Information

## Figures and Tables

**Figure 1:**
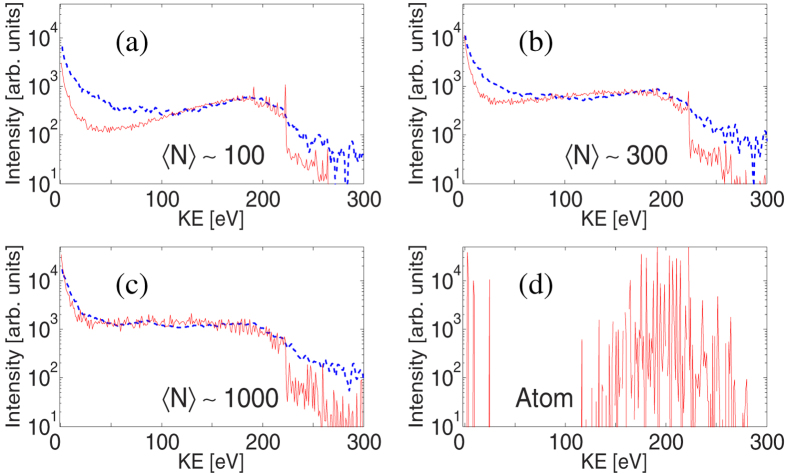
Experimental (dashed blue) and theoretical (solid red) electron energy spectra of Ar clusters, with average sizes of (**a**) 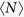
~100, (**b**) 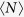
~300, and (**c**) 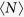
~1000, recorded at the XFEL photon energy of 5 keV at a peak fluence of ~50 *μ*J/*μ*m^2^. As a reference, panel (**d**) shows a theoretical spectrum for single Ar atoms irradiated at the same conditions. Characteristic lines between 100–300 eV(em dash) and below 50 eV correspond to LMM and LLM Auger electrons, respectively.

**Figure 2:**
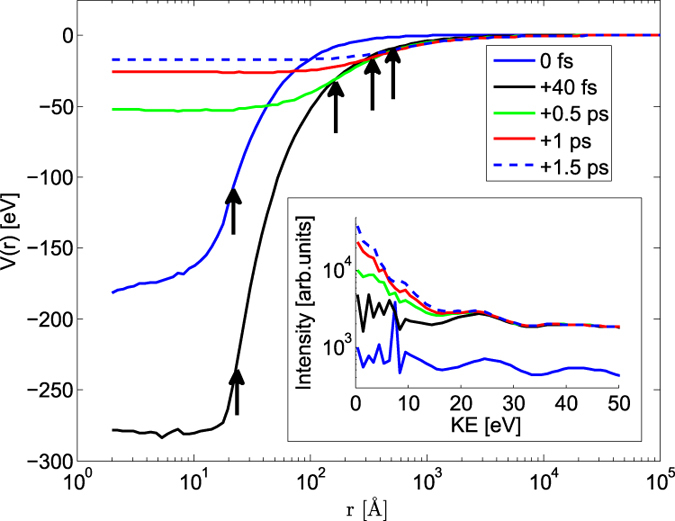
Time evolution of trapping potential 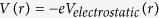 within Ar_1000_ clusters irradiated with a 5 keV pulse at a peak fluence of 50 *μ*J/*μ*m^2^. Arrows indicate the radius *R*_*c*_ of the Ar system, calculated as 
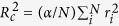
, where *r*_*i*_ is the distance of Ar atoms from the center of the cluster, *α* = 5/3. Inset: volume integrated theoretical electron spectra for various times, 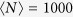. Smoothing was used at energies above 10 eV. The low energy plateau feature is formed during the pulse while the plasma emission peak develops during hundreds of femtoseconds after the pulse. The 10 fs FWHM Gaussian pulse is centered at *t* = 0 fs.

**Figure 3:**
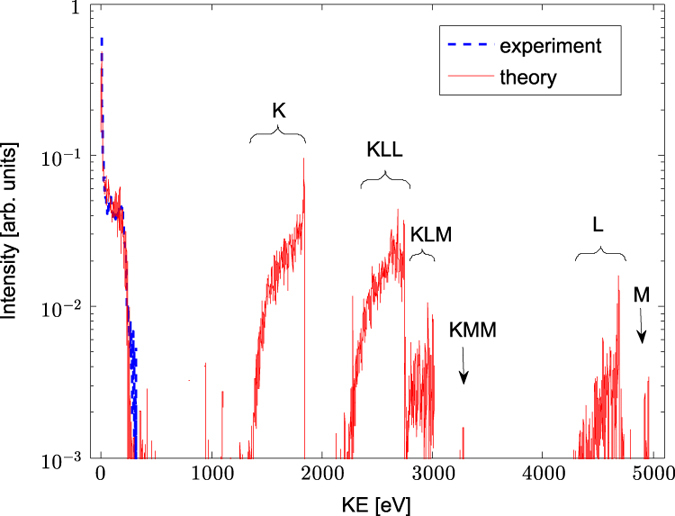
Volume integrated theoretical electron spectrum of Ar clusters of the average size of 1000 atoms plotted for the entire energy range of the ejected electrons. The photon energy is 5 keV and the peak fluence is 50 *μ*J/*μ*m^2^.

**Figure 4:**
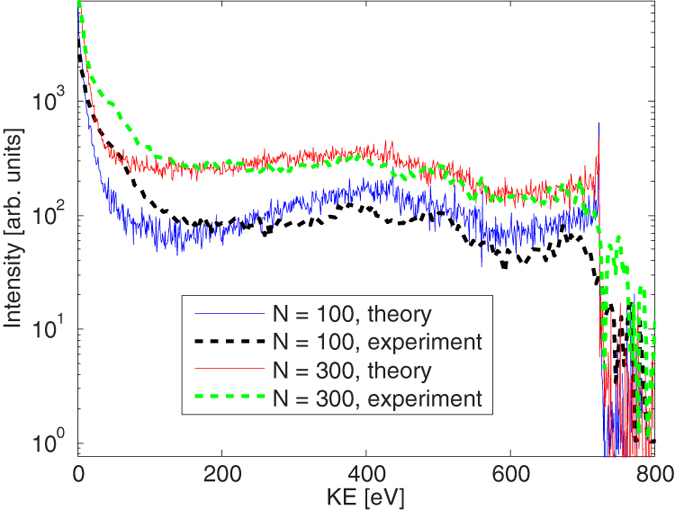
Volume integrated theoretical electron spectrum of x-ray irradiated Xe clusters of the average size of 100 and 300 atoms and the corresponding experimental results within the measured energy range. The photon energy is 5.5 keV and the peak fluence is 45 *μ*J/*μ*m^2^.
