# Supplementary material for: Nanoplasma Formation by High Intensity Hard X-rays
Source: Sci Rep. 2015 Jun 16;5:10977. doi: 10.1038/srep10977 (PMC4468420; doi:10.1038/srep10977)
Supplement: Supplementary Information [file srep10977-s1.pdf]

Supplementary Information for
Nanoplasma Formation by High Intensity Hard X-rays

T. Tachibana, Z. Jurek, H. Fukuzawa, K. Motomura, K. Nagaya, S. Wada, P. Johnsson, M. Siano, S. Mondal, Y. Ito, M. Kimura, T. Sakai, K. Matsunami, H. Hayashita, J. Kajikawa, X.-J. Liu, E. Robert, C. Miron, R. Feifel, J. P. Marangos, K. Tono, Y. Inubushi, M. Yabashi, S.-K. Son, B. Ziaja, M. Yao, R. Santra, and K. Ueda

Experimental details

The XFEL beam was focused by a Kirkpatrick-Baez (KB) mirror system to a focal size of ∼1 μm (FWHM) in diameter. The rare-gas clusters were prepared by adiabatic expansion of the rare gas (Ar or Xe) through a 250 μm nozzle at room temperature. The stagnation pressures were 0.43, 0.67 and 1.12 MPa for Ar and 0.14 and 0.21 MPa for Xe, and the averaged cluster size $\langle N \rangle$ were estimated to be ∼100, ∼300 and ∼1000 for Ar and ∼100 and ∼300 for Xe, respectively, according to the well-known scaling low [32, 33]. If we use more recent work [40] for size estimation, the estimated cluster size becomes ∼20% smaller for the cluster size of 100, but does not change for the larger sizes. The pulsed gas jets were skimmed by two skimmers placed at 20 and 400 mm from the nozzle and introduced to the focal point of the XFEL pulses in an ultrahigh-vacuum reaction chamber [48, 49]. The inner diameters of the first and second skimmers were 0.5 and 2 mm, respectively. The interaction volume defined by the overlap of the XFEL beam and the cluster beam had roughly a cylindrical shape of ∼1 μm in diameter and ∼2 mm (i.e., the diameter of the cluster beam) along the XFEL beam. The photon energy was 5 keV for Ar and 5.5 keV for Xe. The repetition rate of the XFEL pulses was 10 Hz. The pulse width was estimated to be 10 fs (FWHM). The relative x-ray pulse energy passing through the interaction point was measured shot-by-shot by a p-intrinsic-n (PIN) photodiode. The shot-by-shot pulse energy fluctuation was ±25% (50% FWHM). The absolute peak fluence at the interaction point was estimated by measuring the charge distribution of atomic Ar ion yields. The resulting value was ∼50 μJ/μm^2 [48, 49], corresponding to a peak intensity of ∼5×10^{17} W/cm^2, for both Ar and Xe cluster experiments.

Electrons were accelerated towards a position-sensitive detector consisting of a set of micro channel plates (MCPs) followed by a phosphor screen, and recorded using a CCD camera synchronized to the arrival of the FEL pulse in the interaction chamber. A 200-ns electrical gate pulse was applied to the back of the MCPs in order to suppress the influence of dark counts on the detector. The distance between the source point and the MCPs was shorter (112 mm) than in usual VMI configurations [41–43]. This short bore configuration allows us to detect high energy electrons. However, due to the short distances, trajectories of some electrons become almost parallel to the slanted capillaries of MCPs, resulting in low detection efficiencies. Such reduced efficiencies were compensated in the analysis. These corrections were, however, almost negligible in the electron energy spectra discussed in the present paper, whereas they strongly influenced to the angular distribution data. In the Ar cluster experiment, the extraction electric field was set to 213 V/mm at the focal point so that electrons with kinetic energies up to 320 eV could be detected. For Xe clusters, the extraction electric field was set to 643 V/mm so that the electrons with kinetic energies

up to 960 eV could be detected. The measured two-dimensional (2D) projection, after corrections of the position sensitivity described above, allows the three-dimensional (3D) momentum distribution of the ejected electrons to be obtained using the inverse Abel transformation. Note that we focused in our experiment on a limited range of electron energies (< 320 eV for Ar and < 960 eV for Xe), since our main focus is on nanoplasma formation. In such VMI measurements, high-energy electrons beyond the observation range give rise to false peaks at the edge of the image. We compensated these contributions knowing the entire electron energy distributions from theory (see Fig. 3) and performing a series of electron trajectory simulations.

Modelling

For the theoretical modelling, we employed XMDYN [44], our tool developed for modelling finite systems such as clusters irradiated by XFEL. It combines a Monte Carlo description of ionizations with the efficient classical molecular dynamics treatment of particle dynamics (atoms, atomic ions and free electrons). The atomic bound electrons and the ejected free electrons are treated differently. The configurations (occupancies of the atomic orbitals) of electrons bound in the individual atoms are tracked. The electron configurations can change due to outer- and inner-shell photoionization and inner-shell relaxation events (Auger and fluorescence decays). The probability of these processes depends on the corresponding cross sections or rates calculated by the XATOM code [46]. These atomic parameters for all possible transitions for all electron configurations are then collected in a table. In our study, the Monte Carlo algorithm was used to determine the atomic processes occurring during a time step.

The real space dynamics of the classical free electrons, released during an ionization event, and the dynamics of atoms and atomic ions are driven by the Coulomb forces among all charged particles and calculated by numerical integration of Newton equations (Molecular Dynamics). We use a multiple-time-step version of the position Verlet algorithm [50, 51]. The van der Waals interactions between atoms are neglected as the much stronger Coulomb forces dominate the charge dynamics. During electron-ion (atom) collisions secondary (electron impact) ionizations may occur. They are modeled with atomic binary-encounter-Bethe (BEB) cross sections [47], supplied with orbital energy parameters calculated with XATOM. Impact ionization is treated differently than an atomic inner-shell event. When a free electron approaches an ion, its trajectory is extrapolated back to the infinite distance from the point of the closest approach to extract the impact parameter and kinetic energy values for the BEB cross section. Secondary ionization occurs if the impact parameter is smaller than the radius assigned to the calculated cross section [52]. Recombination is taken into account by letting the classical electrons be captured by the ions in a process that corresponds to three- (or many-) body recombination. Three-body recombination is the predominant recombination process in dense media. In order to obtain the initial coordinates of the atoms in real space, we applied a simple energy minimization scheme to a large system of randomly distributed Ar atoms interacting with van der Waals forces [53]. After the energy minimum had been reached, we 'cut out' the inner part of the system, containing the required number of atoms.

In the evaluation process all escaped electrons contributed to the signal that was compared to the experimental data.

The XFEL pulse intensity distribution is spatially non-uniform within the XFEL beam and within the interaction volume defined by the intersection of the beam and the jet. Therefore the measured signal contains contributions from a wide range of fluences from zero to the peak fluence. Thus,

the calculations must include volume integration. This requires calculating spectra for fixed fluence values, and then summing them up with weights determined by the corresponding real–space volume elements. In the simulations, we assumed a Gaussian transversal beam profile. However, we should note that there is a strong indication [31] for the existence of a low fluence halo around the high fluence center of a focused XFEL beam. Depending on the details, a non-negligible signal contribution can originate from such a halo. As we had no such information about the beam conditions in our case, we consistently used the Gaussian profile parametrized with the nominal values for the construction of the volume-integrated signals. A single XMDYN run yields one specific realization of the time evolution of the cluster affected by many stochastic events. A spectrum based on one realization is rather noisy. The statistics can be improved by averaging over the outcome of several individual runs. Note that a lower intensity signal always converges more slowly, i.e. it is noisier than a higher intensity signal. Further, the experimental log-normal distribution of the cluster size was also taken into account based on calculations performed for different cluster sizes. All trajectories were propagated up to 1.5 ps after the pulse. The simulations are getting computationally more expensive with larger cluster sizes. Therefore we chose a combination of the number of cluster sizes, number of fluence values and number of realizations per fluence value in a way that the calculations were feasible and the statistics of the yielded spectra were reasonably good at the same time. We considered 10 cluster sizes from $N = 30$ to $N = 1000$, chose 20 fluence values and from 250 (smallest cluster case) to 3 (largest cluster case) realizations per fluence value. We note that size averaging did not introduce significant changes in the spectra.

References

[50] Barash, D., Yang, L., Qian, X. & Schlick, T. Inherent speedup limitations in multiple time step/particle mesh ewald algorithms. *J. Comput. Chem.* **24,** 77–88 (2003).

[51] Qian, X. & Schlick, T. Efficient multiple-time-step integrators with distance-based force splitting for particle-mesh-Ewald molecular dynamics simulations. *J. Chem. Phys.* **116,** 5971–5983 (2002).

[52] Jurek, Z., Faigel, G. & Tegze, M. Dynamics in a cluster under the influence of intense femtosecond hard X-ray pulses. *Eur. Phys. J. D* **29,** 217–229 (2004).

[53] Coasne, B., Jain, S. K. & Gubbins, K. E. Adsorption and dynamics of argon in porous carbons. *Eur. Phys. J. Special Topics* **141,** 121–125 (2007).
